# Tjalma Syndrome: A Rare Autoimmune Cause of Multisystem Serositis

**DOI:** 10.7759/cureus.105188

**Published:** 2026-03-13

**Authors:** Aaditya Kodamanchile, Sowmya Ekhelikar, Ahmad Kamal Azri AbiMusaAsa'ari, Moustafa Aboushehata, Nawaid Ahmad

**Affiliations:** 1 Department of Cardiology, University Hospitals of North Midlands NHS Trust, Stoke-on-Trent, GBR; 2 Department of Cardiology, Royal Liverpool University Hospital, Liverpool, GBR; 3 Department of Respiratory Medicine, University Hospitals of North Midlands NHS Trust, Stoke-on-Trent, GBR; 4 Department of Pulmonology, University Hospitals of North Midlands NHS Trust, Stoke-on-Trent, GBR; 5 Department of Respiratory and Acute Medicine, Shrewsbury and Telford Hospital NHS Trust, Shrewsbury, GBR

**Keywords:** elevated ca-125, pseudo-pseudo meigs' syndrome, serositis, systemic lupus erythematosus, tjalma syndrome

## Abstract

Tjalma syndrome is a rare manifestation of systemic lupus erythematosus (SLE) characterized by pleural effusion, ascites, and elevated cancer antigen 125 (CA-125) levels in the absence of ovarian malignancy. We report the case of a woman in her 50s who presented with recurrent pleuritic chest pain, dyspnea, peripheral edema, ascites, and constitutional symptoms. Initial investigations were inconclusive, resulting in repeated admissions and multidisciplinary referrals. Subsequent immunological testing confirmed SLE. Given the constellation of serositis and elevated CA-125, a diagnosis of Tjalma syndrome was established. Treatment with immunosuppressants such as corticosteroids, hydroxychloroquine, and azathioprine resulted in symptomatic improvement. However, the disease course was complicated by constrictive pericarditis requiring pericardiectomy and later inflammatory arthritis requiring escalation of immunosuppression. This case highlights the importance of considering autoimmune etiologies in patients with unexplained multisystem effusions and elevated tumor markers, thereby avoiding misdiagnosis and unnecessary oncological interventions.

## Introduction

Tjalma syndrome, also termed pseudo-pseudo Meigs’ syndrome (PPMS), represents a rare but diagnostically consequential manifestation of systemic lupus erythematosus (SLE), characterized by the triad of ascites, pleural effusion, and elevated serum cancer antigen 125 (CA-125) levels in the absence of an ovarian neoplasm. First described by Wiebren A.A. Tjalma in 2005 [1], the syndrome exemplifies the serosal involvement of SLE and underscores the interface between systemic inflammation and oncological mimicry. The combination of significant serous effusions and markedly raised tumor markers frequently triggers an urgent malignancy workup, often prioritizing gynecological cancer in the differential diagnosis and exposing patients to potentially avoidable invasive investigations.

Tjalma syndrome sits within the spectrum of disorders related to Meigs’ syndrome [2] and pseudo-Meigs’ syndrome [3], yet it is distinguished by the absence of any pelvic or ovarian tumor. Unlike classical Meigs’ syndrome, where effusions resolve following tumor resection, the pathophysiology of Tjalma syndrome is driven by immune-mediated serositis and mesothelial activation. Elevated CA-125 levels are believed to reflect inflammatory stimulation of mesothelial cells rather than malignant transformation, correlating with lupus disease activity and systemic cytokine burden [1,4]. This pathobiological mechanism situates the syndrome within the broader cardiovascular and serosal complications of SLE, where pleuro-pericardial and peritoneal inflammation may coexist and contribute to significant morbidity.

Given its rarity and its striking phenotypic overlap with advanced malignancy, Tjalma syndrome remains underrecognized across specialties, including respiratory medicine, cardiology, rheumatology, and acute medicine. Early recognition is essential to prevent unnecessary surgical intervention and to facilitate timely immunosuppressive therapy targeting the underlying inflammatory cascade. Beyond its diagnostic implications, the condition highlights the systemic consequences of uncontrolled autoimmune inflammation and the importance of multidisciplinary evaluation in complex serosal presentations. This case report delineates the clinical trajectory of our patient, the diagnostic challenges encountered, and the approach that ultimately led to the diagnosis of Tjalma syndrome.

## Case presentation

**Figure 1:**
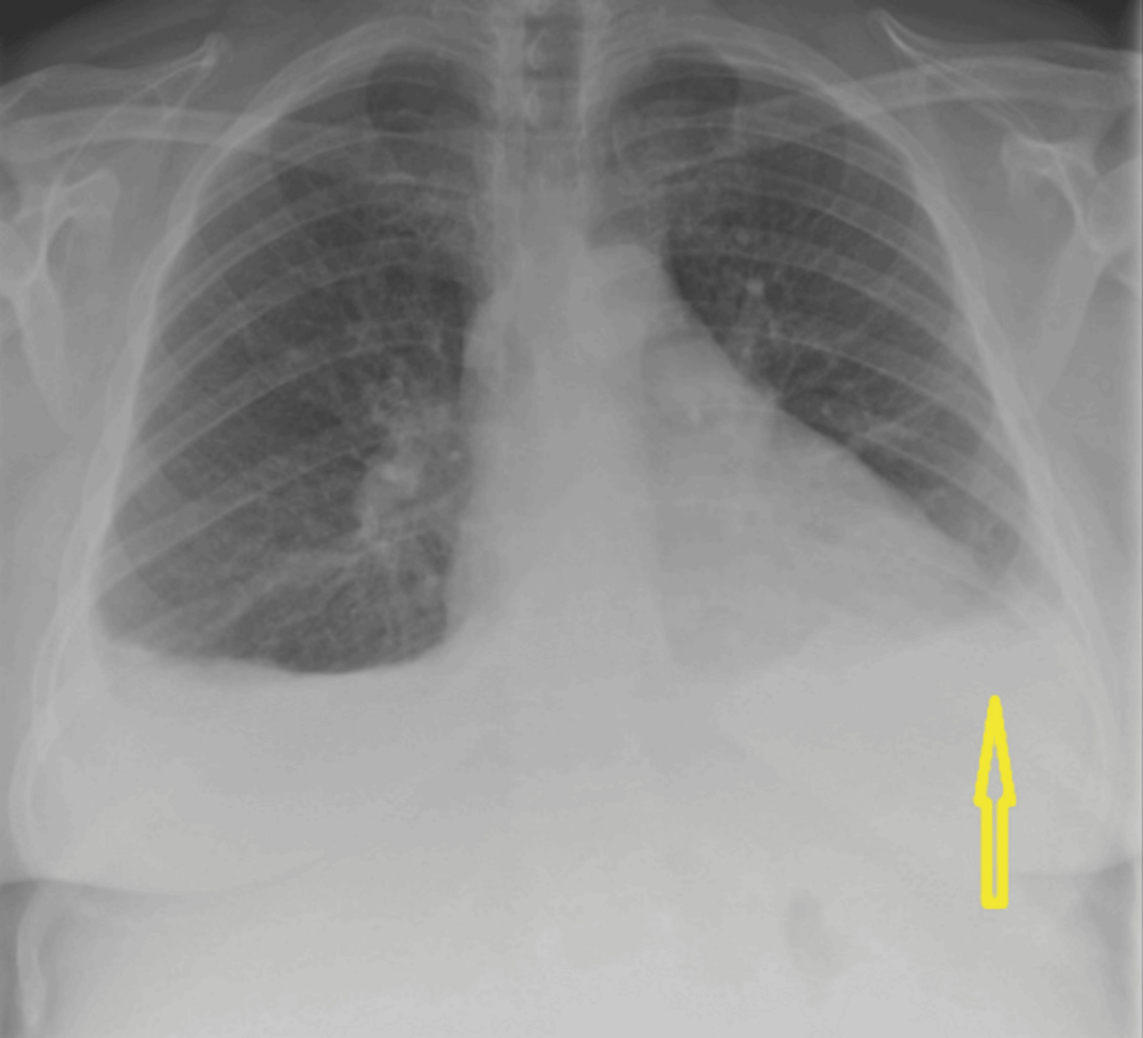
Chest X-ray showing pleural effusion (yellow arrow)

A woman in her 50s initially presented with recurrent left-sided pleuritic chest pain; her blood tests showed elevated inflammatory markers, so she was treated for possible pneumonia (Figure 1).

**Figure 2:**
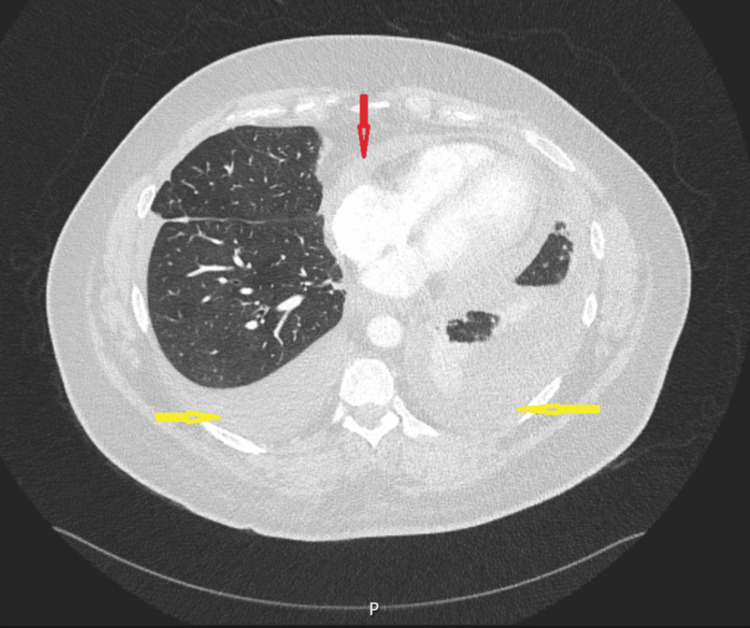
CT scan of the thorax showing bilateral pleural effusions (yellow arrows) and pericardial effusion (red arrow)

She re-presented a few days later with persistent symptoms. D-dimer levels were elevated; therefore, a CT pulmonary angiogram was performed, which excluded pulmonary embolism but demonstrated bilateral pleural effusions and a small pericardial effusion. These findings were attributed to infection, and the patient was discharged following antibiotic therapy (Figure 2).

**Figure 3:**
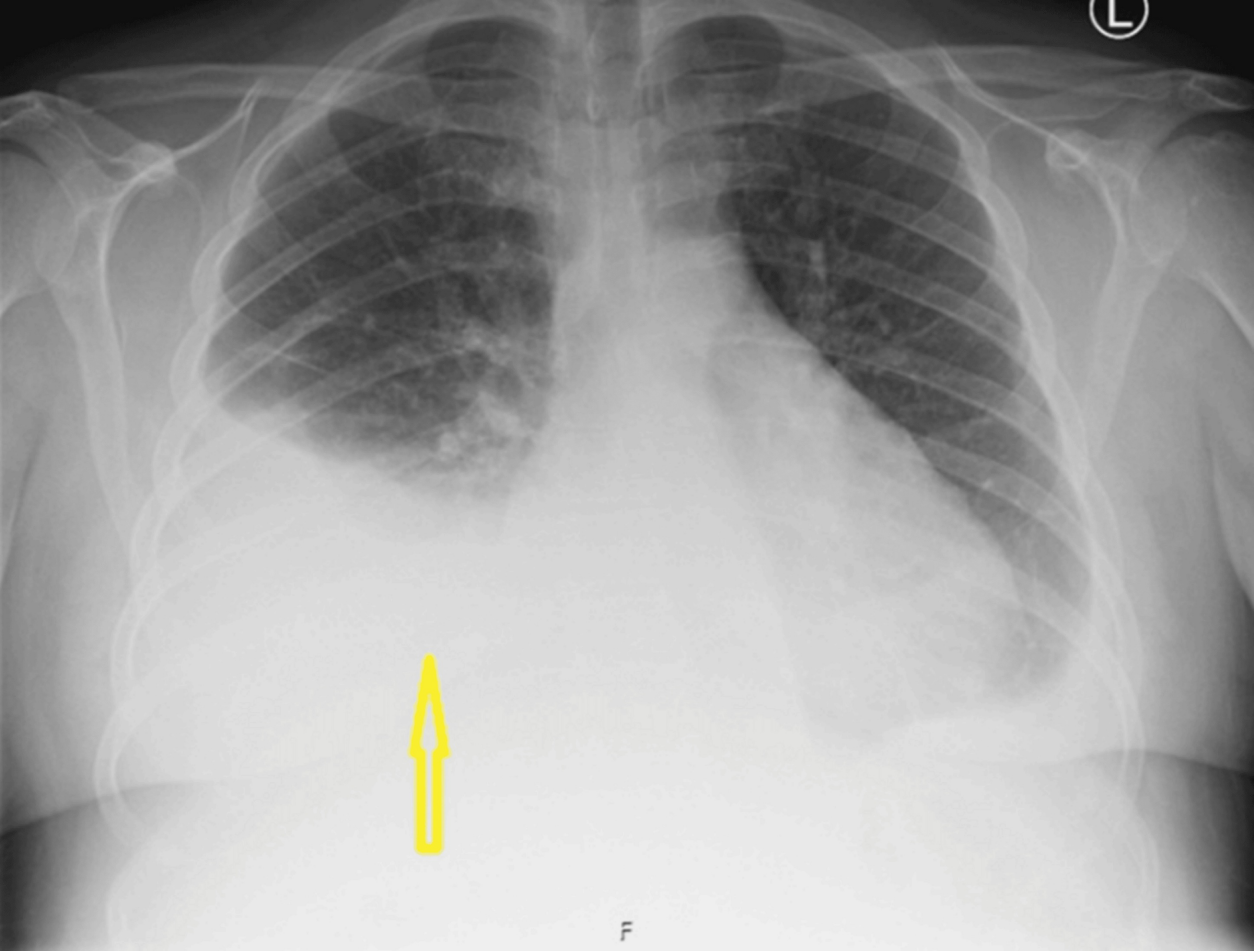
Repeat chest X-ray taken a few months later showing persistent pleural effusions (yellow arrow)

A few months later, she re-presented with progressive dyspnea on exertion, dry cough, bilateral pedal edema, abdominal distension, and generalized myalgia following recent travel. On examination, she was afebrile and hemodynamically stable, requiring low-flow oxygen. Laboratory tests showed mildly elevated inflammatory markers. Chest radiography demonstrated persistent bilateral pleural effusions (Figure 3).

**Figure 4:**
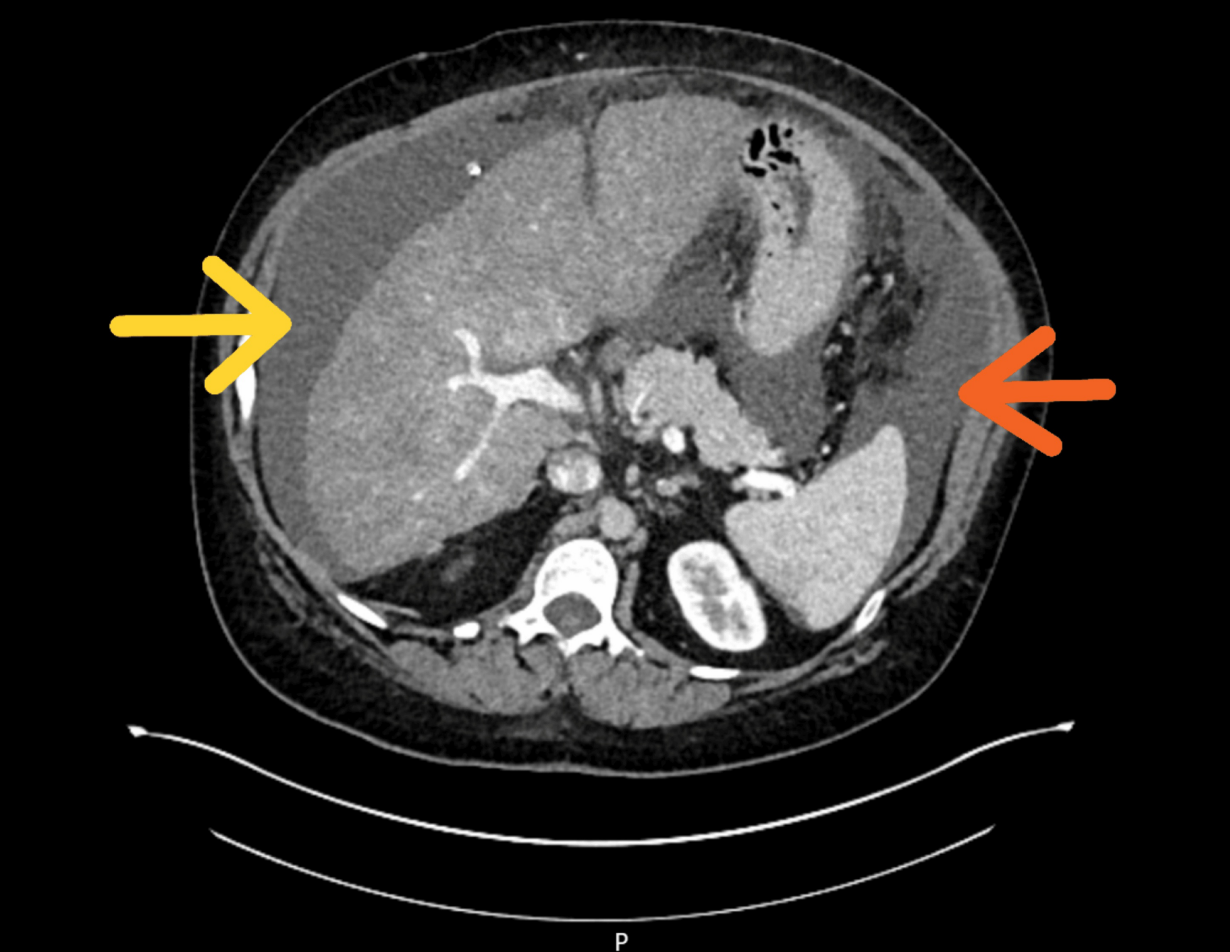
CT scan of the abdomen and pelvis showing ascites and small volumes of fluid in the perihepatic (yellow arrow) and perisplenic areas (orange arrow)

She was treated empirically for infection and possible heart failure with antibiotics and diuretics, with transient symptomatic improvement. An outpatient CT scan of the thorax, abdomen, and pelvis was requested, which revealed persistent small bilateral pleural effusions, pericardial effusion, mediastinal lymphadenopathy, a bulky uterus with a necrotic-appearing lesion, ascites, and small volumes of fluid in the perihepatic and perisplenic areas (Figure 4).

Serum CA-125 levels were markedly elevated (Table 1).

**Table 1:** CA-125 levels CA-125 levels were obtained following CT of the abdomen and pelvis and were subsequently repeated during further follow-up. CA-125, cancer antigen 125

Measurement	Value (U/mL)	Normal range
Initial measurement	113	0-35
Follow-up measurement	447	0-35

These findings prompted referrals to respiratory, cardiology, gynecology, and rheumatology services. Multiple diagnostic thoracenteses were performed, all of which confirmed an exudative pleural effusion with inflammatory predominance and no evidence of malignancy or tuberculosis, supporting a nonmalignant inflammatory process. Gynecological investigations, including pelvic ultrasound, hysteroscopy, and biopsy, excluded malignancy. Microscopy confirmed mostly normal endometrial tissue with occasional fragments that have thicker-walled vessels, which raised the possibility of a benign endometrial polyp. Cardiac MRI demonstrated inflammatory pericarditis.

Further rheumatological assessment revealed alopecia, possible Raynaud’s phenomenon, persistent lymphopenia, strongly positive antinuclear antibodies (1:320, homogeneous pattern), and markedly elevated anti-double-stranded DNA (anti-dsDNA) titers, confirming a diagnosis of SLE (Table 2).

**Table 2:** Autoimmune and inflammatory marker profile Initial serologic evaluations, including ANA and ANCA, yielded negative results. However, repeat testing later identified ANA and anti-dsDNA positivity, demonstrating that persistent clinical suspicion warrants longitudinal follow-up and re-testing. ANA, antinuclear antibody; ANCA, anti-neutrophil cytoplasmic antibody; anti-dsDNA, anti-double-stranded DNA; ENA, extractable nuclear antigen

Test	Day 1	Day 104	Day 143	Normal range
ANA titer	1:80 (speckled)	1:320 (homogeneous)	1:320 (homogeneous)	<1:80
Anti-dsDNA (ELiA)	Not tested	368.0 IU/mL	>379 IU/mL	0-10 IU/mL
Other screenings (anti-ENA and anti-liver antibodies)	Not tested	Negative	Negative	Negative

**Figure 5:**
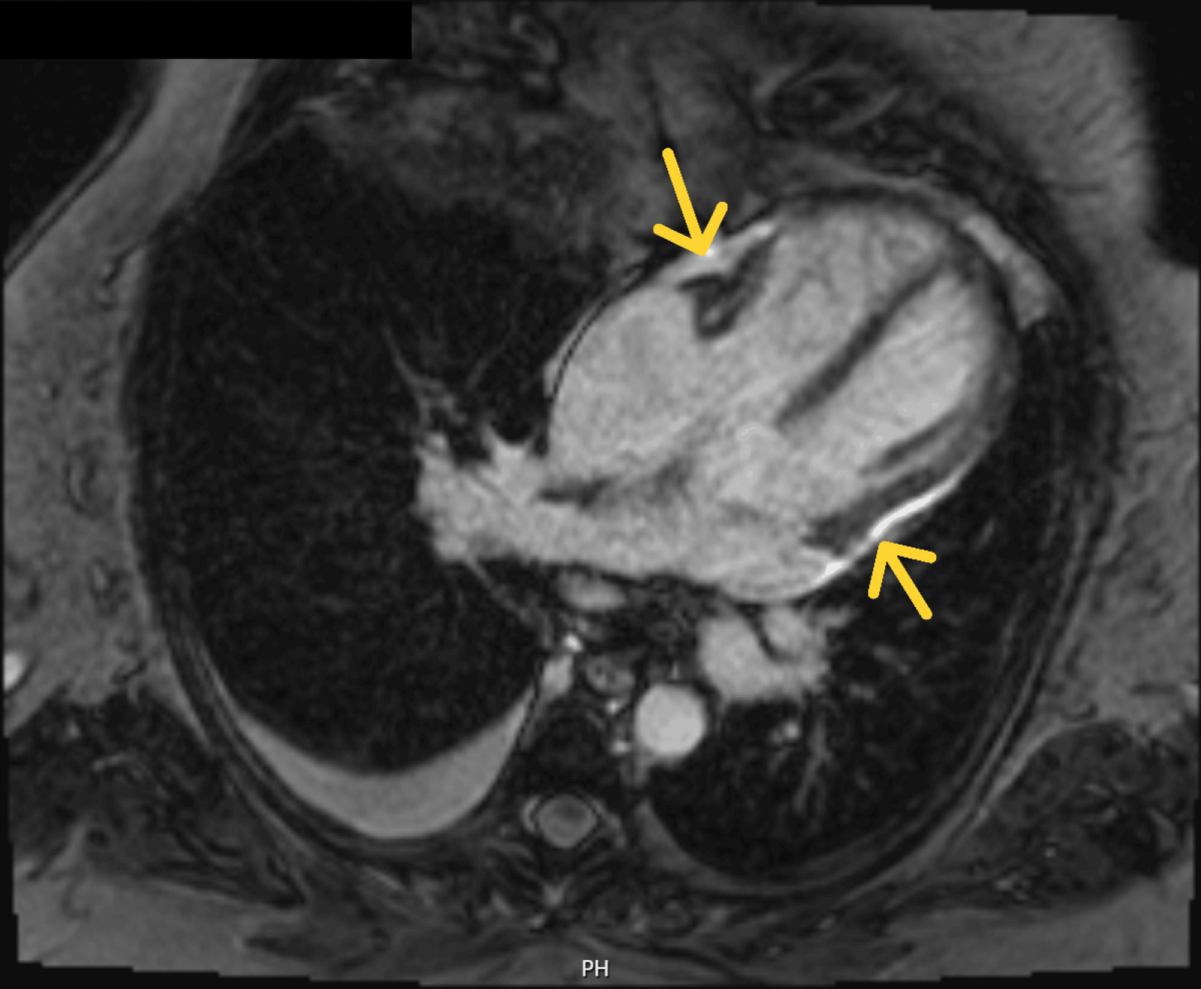
Cardiac MRI apical four-chamber view showing pericardial thickening and late gadolinium enhancement of the pericardium (yellow arrows)

**Figure 6:**
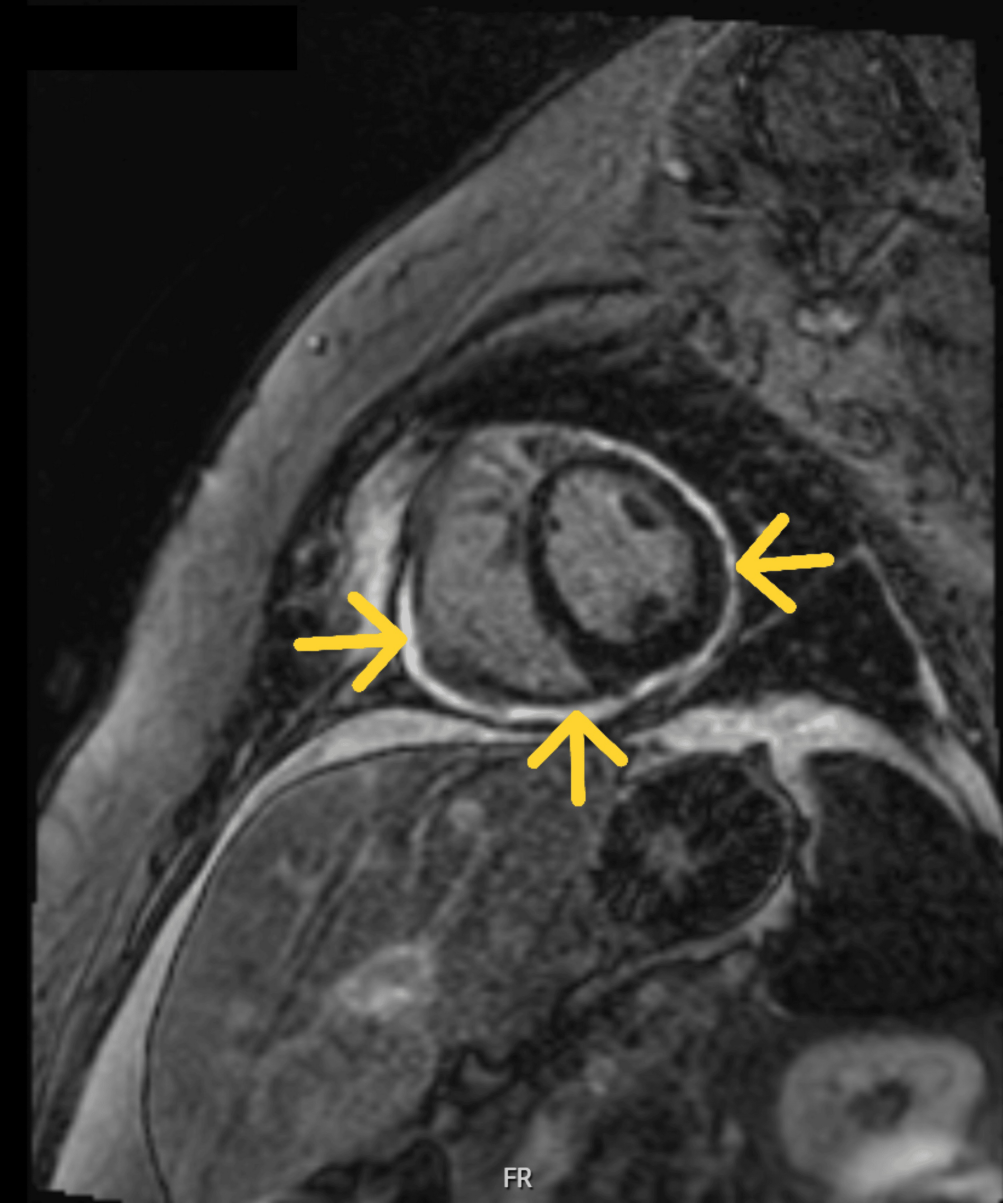
Cardiac MRI parasternal short-axis view showing pericardial thickening and late gadolinium enhancement of the pericardium (yellow arrows)

Given the presence of pleural effusion, pericardial effusion, ascites, and elevated CA-125, a diagnosis of Tjalma syndrome was made. The patient was treated with corticosteroids and hydroxychloroquine, with initial clinical improvement. However, she subsequently developed constrictive pericarditis (Figure 5, Figure 6).

She required surgical pericardiectomy, after which her serositis resolved. Subsequently, inflammatory arthritis became the predominant manifestation, necessitating escalation of immunosuppressive therapy with methotrexate and intramuscular corticosteroids.

## Discussion

Tjalma syndrome, also referred to as PPMS, is a rare clinical entity characterized by the triad of pleural effusion, ascites, and elevated CA-125 in the absence of an ovarian or pelvic tumor [1-5]. The syndrome has since been increasingly recognized as a manifestation of SLE [6,7].

Unlike classic Meigs’ syndrome, which is associated with benign ovarian fibromas, or pseudo-Meigs’ syndrome, which involves other pelvic tumors, Tjalma syndrome occurs without an underlying neoplasm. The presence of ascites, pleural effusion, and markedly elevated CA-125 frequently prompts extensive oncological investigations and, in some cases, unnecessary surgical intervention. Increased awareness of this entity is therefore critical to prevent misdiagnosis and inappropriate management.

Pathophysiology

The precise pathophysiological mechanisms underlying Tjalma syndrome remain incompletely understood; however, evidence supports immune-mediated serositis rather than a malignant process. In SLE, immune complex deposition, cytokine release, and complement activation result in chronic inflammation of serosal surfaces, including the pleura and peritoneum. This inflammatory cascade increases capillary permeability, leading to the accumulation of exudative pleural effusions and ascites [7].

The elevation of CA-125 is particularly misleading and central to diagnostic confusion. CA-125 is a high-molecular-weight glycoprotein expressed by mesothelial cells lining the pleura, peritoneum, and pericardium, in addition to Müllerian epithelium [8,9]. Consequently, inflammatory irritation of serosal membranes can lead to substantial elevations in CA-125, sometimes reaching levels comparable to those observed in ovarian malignancy [8,9]. Multiple case reports have demonstrated normalization of CA-125 following immunosuppressive therapy, reinforcing its role as a marker of serosal inflammation rather than malignancy.

Clinical presentation and diagnostic considerations

Patients with Tjalma syndrome can have a wide range of presentations, including progressive dyspnea, abdominal distension, chest discomfort, weight loss, and constitutional symptoms, closely mimicking intra-abdominal malignancy. The condition predominantly affects women, often raising immediate concern for gynecological cancer, as seen in our patient.

Radiological imaging typically demonstrates moderate to large pleural effusions and ascites, without evidence of an ovarian or pelvic mass. Cytological analysis of pleural and ascitic fluid usually reveals exudative effusions without malignant cells, although repeated sampling may be required to confidently exclude malignancy. The absence of neoplastic cells, together with unremarkable pelvic imaging, should prompt consideration of autoimmune etiologies.

Autoimmune serological testing plays a pivotal role in diagnosis. When suspicion for an autoimmune etiology is high, repeated testing may sometimes be necessary, as seen in our case. Elevated antinuclear antibodies, anti-dsDNA antibodies, hypocomplementemia, and other lupus-specific markers support the diagnosis of SLE-associated Tjalma syndrome. Importantly, several reports describe Tjalma syndrome as the initial manifestation of SLE, including cases of juvenile-onset disease, preceding classical clinical features such as arthritis or renal involvement [10,11].

Differential diagnosis

The differential diagnosis of pleural effusion, ascites, and elevated CA-125 is broad and includes ovarian malignancy, peritoneal carcinomatosis, tuberculosis, liver cirrhosis, heart failure, and nephrotic syndrome. Abdominal tuberculosis is a particularly important mimic, as it may present with lymphocyte-predominant exudative ascites and elevated CA-125, especially in endemic regions. Careful microbiological, biochemical, and histopathological evaluation is often required to exclude infectious causes.

Distinguishing Tjalma syndrome from Meigs’ and pseudo-Meigs’ syndromes relies on the absence of a causative ovarian tumor and the presence of autoimmune disease. Failure to recognize this distinction may lead to unnecessary invasive procedures, including diagnostic laparotomy or oophorectomy, with avoidable morbidity [11].

Management and outcomes

Management of Tjalma syndrome mainly involves treating the underlying inflammatory and autoimmune process. Systemic corticosteroids are the cornerstone of therapy and typically result in rapid clinical improvement, resolution of pleural effusions and ascites, and normalization of CA-125 levels [6]. In refractory or severe cases, additional immunosuppressive agents such as azathioprine, mycophenolate mofetil, cyclophosphamide, or intravenous immunoglobulin may be required, particularly in the presence of concurrent major organ involvement [11].

Therapeutic drainage of pleural effusions or ascites may be necessary for symptomatic relief but should be regarded as supportive rather than definitive. Recurrence of effusions is common if immunosuppressive therapy is delayed or inadequate. Recurrent pericarditis can lead to constrictive pericarditis through a process of chronic inflammation, resulting in fibrous thickening, scarring, and, in some cases, calcification of the pericardium, requiring surgical pericardiectomy, as seen in our patient.

Overall, the prognosis of Tjalma syndrome is favorable when appropriately recognized and treated. Unlike malignant causes of ascites and pleural effusion, the condition does not carry an adverse oncological prognosis. Long-term outcomes depend primarily on effective control of the underlying autoimmune disease.

## Conclusions

Tjalma syndrome underscores the limitations of CA-125 as a tumor marker and highlights the importance of interpreting laboratory results within the appropriate clinical context. Clinicians should maintain a high index of suspicion for autoimmune causes in patients presenting with unexplained serositis and elevated CA-125, particularly when imaging and invasive testing fail to demonstrate malignancy. Early recognition can prevent unnecessary invasive procedures, reduce patient anxiety, and allow timely initiation of immunosuppressive therapy, resulting in excellent clinical outcomes.
